# Applications of Blockchain and Smart Contracts to Address Challenges of Cooperative, Connected, and Automated Mobility

**DOI:** 10.3390/s24196273

**Published:** 2024-09-27

**Authors:** Christos Kontos, Theodor Panagiotakopoulos, Achilles Kameas

**Affiliations:** 1 School of Science and Technology, Hellenic Open University, 18 Parodos Aristotelous Street, 26335 Patras, Greece; kontoschr@gmail.com (C.K.); kameas@eap.gr (A.K.); 2 Business School, University of Nicosia, 46 Makedonitissis Street, 2417 Nicosia, Cyprus

**Keywords:** cooperative, connected, and automated mobility, blockchain, smart contracts, internet of things, internet of vehicles

## Abstract

Population growth and environmental burden have turned the efforts of cities globally toward smarter and greener mobility. Cooperative and Connected Automated Mobility (CCAM) serves as a concept with the power and potential to help achieve these goals building on technological fields like Internet of Things, computer vision, and distributed computing. However, its implementation is hindered by various challenges covering technical parameters such as performance and reliability in tandem with other issues, such as safety, accountability, and trust. To overcome these issues, new distributed and decentralized approaches like blockchain and smart contracts are needed. This paper identifies a comprehensive inventory of CCAM challenges including technical, social, and ethical challenges. It then describes the most prominent methodologies using blockchain and smart contracts to address them. A comparative analysis of the findings follows, to draw useful conclusions and discuss future directions in CCAM and relevant blockchain applications. The paper contributes to intelligent transportation systems’ research by offering an integrated view of the difficulties in substantiating CCAM and providing insights on the most popular blockchain and smart contract technologies that tackle them.

## 1. Introduction

The constantly growing population in urban settlements is the source of many problems for their citizens such as heavy traffic, long commuting times, air and noise pollution, expansion of transport infrastructure, and increase in vehicles [1]. In recent years, the spread of advanced technologies and especially the integration of Internet of Things (IoT) in urban transportation infrastructures, allowed the development of smarter and greener mobility systems and applications. Smart mobility is an umbrella term used to describe and promote the disruptive changes related to the automation, digitalization, and economics of transport infrastructure [2].

Smart and green mobility has three goals [3]: sustainability, safety, and cost-effectiveness. In order to meet them, it encompasses a rich variety of innovative IoT-driven solutions: smart parking systems, smart logistics, shared mobility, integrated ticketing systems, route optimization, autonomous driving, smart payment systems, cooperative vehicle awareness, route optimization, traffic management, accident detection, and road anomalies detection [1,4,5,6].

One of the most prominent emerging application areas of smart mobility is Cooperative, Connected, and Automated Mobility (CCAM), which allows vehicles to interact with each other and transportation infrastructures, enabling real-time data collection and analysis for coordinated action [7]. It has generated significant attention among researchers, industry, and governments as it is expected to unveil disruptive opportunities for societies and economies [8]. Connected and automated vehicles (CAVs) have the ability to address traffic congestion in a cooperative manner [9] and the potential to remove risk factors associated with human driving errors and therefore to reduce the number of road accidents, while improving the functionality of transport systems [10].

However, safe and reliable operation of CAVs still needs considerable progress, testing for building acceptance and trust and regulatory approval for their commercial availability [11]. In order to deliver on their promise, CAVs need to overcome a range of shortcomings and limitations, such as those related to currently used sensing technologies [12] and centralized computing architectures [13]. This is exacerbated by the complexity and scale of vehicular IoT networks, which make the application of CCAM in smart cities more difficult and hinder large-scale urban deployments. Additionally, various scholars argue that technological issues of CCAM create important ethical, legal, and societal considerations (e.g., [14]). To overcome these issues, new technologies are explored based on distributed and decentralized approaches. Toward this direction, the emergence of blockchain technologies and smart contracts has become a key enabler of distributed systems and IoT infrastructures in urban transportation.

The process of selecting/developing and applying appropriate blockchain and smart contracts mechanisms in CCAM solutions predominantly relies on the challenge that needs to be solved. Although several works present important challenges of CCAM and propose blockchain-based and other mechanisms to deal with them, an inclusive taxonomy is still missing. This is because these challenges are examined from specific perspectives directly related to different applications of autonomous driving in smart cities. To fill this gap, our work aims to piece together a more comprehensive understanding of CCAM challenges by drawing up a detailed inventory of theirs and build on this to explore the most prominent blockchain and smart contracts mechanisms to address them. Table 1 displays recent surveys on CCAM challenges and demonstrates the novelty of our paper.

The contribution of this work is threefold. First, it presents a comprehensive list of CCAM challenges classified into three categories: technical, social, and ethical (see Table 1). In addition, the importance of these challenges and the impact they have in the acceptance of CCAM in mobility is discussed. Second, it identifies existing methodologies and solutions to address these challenges based on blockchain and smart contracts. Third, we make a critical review of these methodologies eliciting qualitative and quantitate features to create a framework for comparing different methods’ performance for each challenge.

## 2. Background

The purpose of this section is to present the background knowledge around the basic building blocks of this work.

### 2.1. Vehicular Ad Hoc Networks and CCAM

Vehicular Ad hoc Networks (VANETs) are a subclass of unstructured Mobile Ad hoc Networks (MANETs) in which the nodes of the network are the vehicles that communicate both with each other and with the base stations [21]. The vehicles participate in the network either as routers or as wireless access points and move continuously. They support three types of communication: in-vehicle communication, unstructured communication with other vehicles and with base stations (ad hoc), and infrastructure communications (infrastructural) [22].

The nodes in VANETS are located exclusively on vehicles, and their topology is very dynamic. Their routes are mostly predefined as the vehicles move exclusively on the road network [23]. Vehicles have varied capabilities in terms of processing power, autonomy (energy supply), and data storage, but they require a continuous supply of high bandwidth for efficient operation [24]. These nodes move at very high speeds and change their position instantaneously and continuously. At the same time, their connection to both the neighboring vehicles and the central network is interrupted and connected continuously.

The VANETs are also a subnetwork of Internet of Vehicles (IoV) since the latter contain other networks such as the communication network of infrastructures with each other and with the Internet and, finally, the interconnection of all those involved in smart mobility, people, vehicles, things, and the road environment. According to [21], applications of VANETs in smart mobility are divided into two categories:

- Safety applications: collision avoidance, curve speed warning, traffic signal violation, emergency brake lights, pre-collision detection, collision warning, left turn assist, lane change warning.
- Non-safety applications: traffic information, infotainment applications, weather, and points of interest information.

The architecture of VANETs consists of three domains: mobile, infrastructure, and general domain [21]. The mobile domain includes the vehicles with their On-Board Units (OBUs), Mobile Units (MUs) and Application Units (AUs). Roadside Units (RSUs) are network components that are usually located at fixed points along the sides of the road or at specific locations [25]. The RSUs along with the sensors of a smart city, belong to the infrastructure domain. The general domain contains the Internet access and some private infrastructure like SaaS or IaaS providers or content providers. The architecture of VANETs provides communications between mobile nodes (vehicles) and fixed points located along a road [26]. There are two categories of communications [27]:

- Mobile communications: In-Vehicle and Vehicle-to-Vehicle (V2V)
- Fixed node communications: Vehicle-to-Infrastructure (V2I) and Vehicle-to-Broadband Cloud (V2B or V2C)

### 2.2. Blockchain Fundamentals

Blockchain first appeared in 2008 bringing new and unique features to the IT and Internet world that were not possible before such as reliable decentralized information systems without the mediation of a central authority [28]. The original idea was to store in a file called a block the data of the transaction as well as the code of the previous transaction along with a timestamp. Each new block was linked to the previous one using cryptographic techniques, thus forming a chain of blocks. The motivation was to create a digital or virtual currency that would maintain its value without the involvement of any financial or banking central entity. But the key technology that started from this work and that has caught the interest of the research world in the field of IT while at the same time transforming the business world is blockchain.

Blockchain is a chain of transactions in which each transaction is related to the previous transaction. The unique ID of each block is a hash that is created using the hashing algorithm with 256-bit encryption (SHA256) and that is applied to the header of the block. A key feature of this algorithm is that knowing its output, one cannot discover the original data before encryption, while the more possible hashes, the less chance there is of two values creating the same hash value [29].

**Figure 1:**
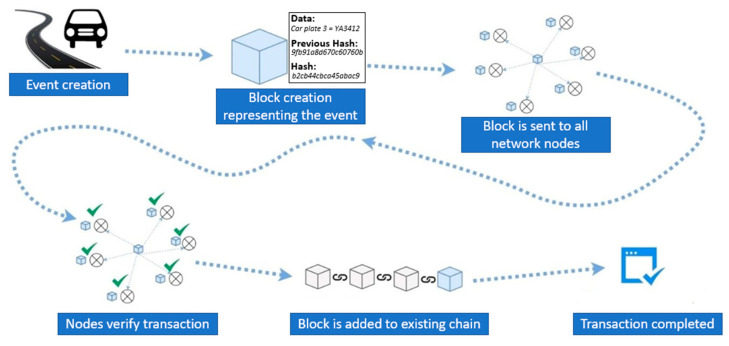
The stages of creating and adding a new block.

Blockchain is a pervasive network consisting of computer nodes connected to the Internet that together maintain an account of transactions performed on the network. A record of each transaction is shared throughout the network, while the approval of the transaction is not done by a central system but by a group of computers participating in the network. These records, which are called blocks, are part of a chain of blocks (blockchain), and each of them is related (referenced) to the previous block. Looking at transportation and CCAM (Figure 1), blockchain has been used in a rich variety of applications, such as forensics [30], sensor security [31], and collective intelligence protection [32].

### 2.3. Smart Contracts

A division in blockchain technology based on two transaction models; the token-based model and the account-based model are presented in [13]. Smart contracts belong to the second model and are placed in the second stage of blockchain development called Blockchain 2.0.

The general definition of smart contracts states that a smart contract consists of a computer program that can automatically execute and apply the terms of a contract [33]. More specifically, a smart contract refers to a computational transaction protocol that executes the terms of a contract, satisfying common contractual terms such as payment, retention, confidentiality, and enforcement of an agreement while at the same time minimizing malicious or accidental disputes as well as the need for trusted intermediaries [34]. Smart contracts are self-executing and incorporate the ownership information of assets, thus overcoming the problem of counterparty trust [35]. By assets, we do not necessarily mean coins but any asset that can be digitized. Thus, we observe that the use of smart contracts as digital money protocols can be applied not only to money but also to a wide variety of digital assets [36]. According to the transactions carried out through conventional financial organizations, many techniques are needed for a payment to be safe and guaranteed, such as live contact, certified mail, and credibility of the contracting party’s credit history. Smart contracts go beyond the above techniques, providing transparency, reliability, and cost reduction due to both the elimination of fraud and the observance of agreements (guarantees) [37].

**Figure 2:**
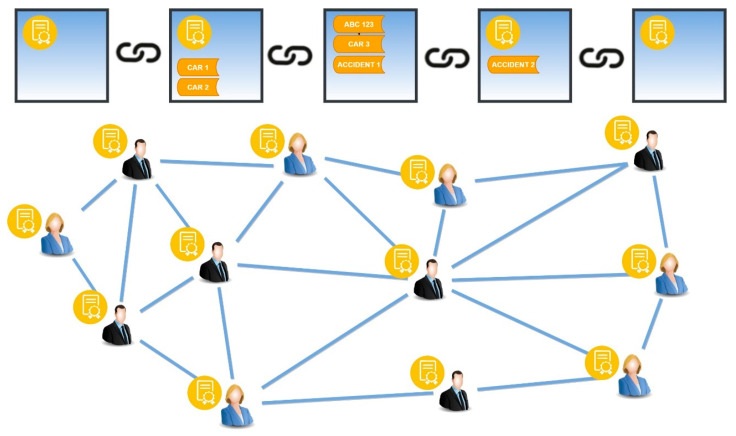
A smart contract is based on blockchain technology.

Smart contracts can help eliminate the need for intermediaries, such as banks or legal professionals, in many types of transactions. They can also increase efficiency, reduce costs, and improve security by eliminating the need for human intervention and reducing the risk of fraud or error [38]. Because smart contracts are stored on a decentralized blockchain, they are immutable and transparent, meaning that once a smart contract is deployed, it cannot be altered or tampered with (Figure 2). This makes smart contracts a secure and trustworthy way to conduct transactions and enforce agreements between parties.

Smart contracts work through four steps [39]. In the first step, the contracting parties should determine the terms of the contract (payment, retention, confidentiality, etc.) as well as the conditions of execution such as the time of execution. Then, once the contractual terms are agreed and finalized, they are converted into programming code. In the second step, the execution of the contract is activated. Execution is triggered by either an event such as an initialization transaction or the collection of information appropriate for the execution of the contract. In the third step, the code of the contract is executed by all the nodes of the network, and if any condition of the contract is satisfied and verified by all the participating nodes of the blockchain network, then the values are transferred according to the initial conditions of the contract. In the fourth step, the agreement (settlement) is performed or the liquidation of the elements that are on the chain, such as, for example, digital currencies. However, in the case of off-chain items such as shares or cash, then their respective accounts are updated

## 3. Method

### 3.1. Research Questions

Based on the research objectives of our study, we formulated the following research questions to examine what current literature includes CCAM challenges and related mechanisms to address them based on blockchain and smart contracts.

RQ1: Which are the most important challenges of CCAM, and how can they be classified?

RQ2: Which solutions utilizing blockchain technologies and smart contracts are used in response of CCAM challenges?

RQ3: How does each blockchain and/or smart contract method perform, and what are its pros and cons?

### 3.2. Research Methodology

We followed a Systematic Literature Review (SLR) approach [40] using well-specified methods to identify, screen, and eventually select research articles directly connected to our study’s research questions. We employed the PRISMA model for our SLR [41], which was conducted at two phases.

**Figure 3:**
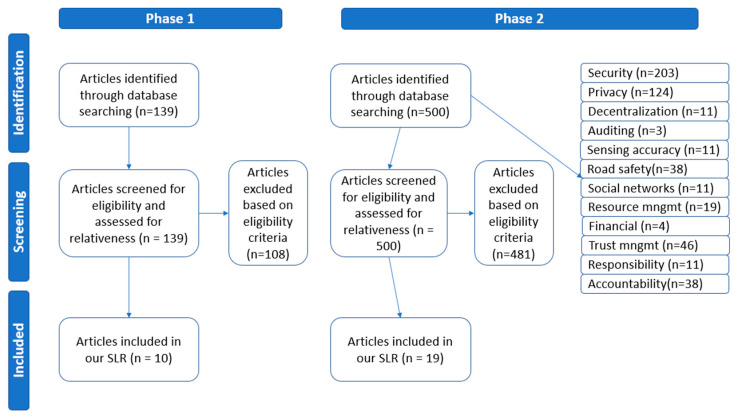
PRISMA review process.

The first phase focused on the challenges of CCAM. Several well-known electronic scientific databases were queried for the literature review. In order to enhance the credibility and integrity of the study, exclusively peer-reviewed journal or conference papers were taken under consideration. We searched the scientific databases IEEE Xplore, Science Direct, and Scopus using the string (“Cooperative” AND “Connected” AND “Autonomous” AND “Vehicles” AND “Challenges”). We also limited publication date between 2018 and 2023. A total of 139 unique articles from the search were screened and assessed based on the title and abstract. We excluded duplicates and non-peer-reviewed journal or conference articles and articles not focusing on surveys about CCAM challenges, reducing the number of relevant articles to 10 (Figure 3). The selected papers were downloaded and read in full. Based on the articles included in our SLR, we elicited 12 major CCAM challenges and categorized them into technical, social, and ethical challenges.

The second phase consists of the literature review for methods that deal with the above challenges in CCAM with the application of blockchain and smart contracts. For the identification of articles addressing this topic, we searched for articles in the same three databases. Our search string was (“VANET” OR “Vehicular Networks” OR (“Cooperative” AND “Connected” AND “Autonomous” AND “Vehicles”) AND “blockchain” OR “smart contract” AND “name of each CCAM challenge”), and we performed 12 searches, one for each CCAM challenge. We excluded duplicates and non-peer-reviewed journal or conference articles, as well as articles not providing empirical data, such as abstracts, editorials, conference summaries, short papers, and book chapters. It is important to mention that our search revealed several articles employing similar methods. In such cases, we included only one article per method in our study, based on the extent of technical details, and the existence of a case study and evaluation. All non-English written articles were also excluded. Nineteen articles met these criteria, as shown in Figure 3. We studied the full texts of these articles and conducted a further backward reference search to learn more about this body of knowledge development.

## 4. Taxonomy of Challenges in CCAM

Autonomous and connected vehicles have significant shortcomings and limitations as well as challenges. For example, autonomous vehicles have limitations in sensing technologies that are not reliable in particular weather and road conditions. Furthermore, integrated AI systems function as a “black box” without a clear explanation of how they work. On the other hand, connected vehicle technologies are completely dependent on messaging to achieve mutual understanding, which will have a big impact when the former gains a lot of penetration on the streets of smart cities [12].

**Figure 4:**
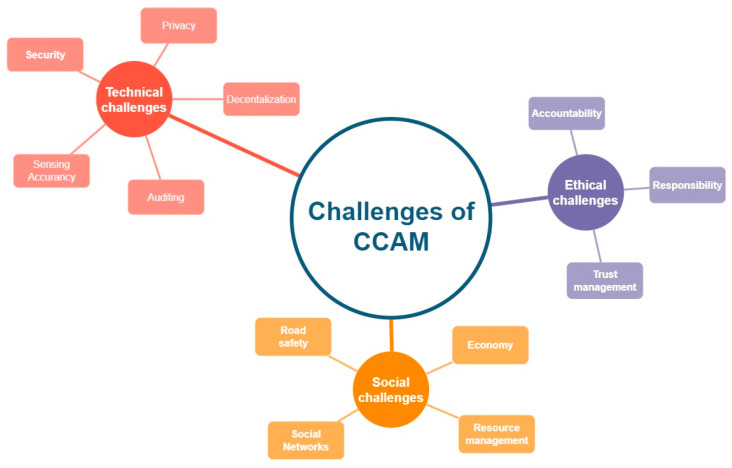
The taxonomy of challenges in CCAM.

Based on the relevant literature, we identified 12 major challenges in CCAM classified in three categories: technical, social, and ethical (Figure 4).

### 4.1. Technical Challenges

Security: Security of data and messages exchanged between vehicles and with RSUs and central infrastructures. Information security and cybersecurity issues such as issues of availability, data integrity, confidentiality, authenticity, and the corresponding attacks.

Privacy: Protection of the data of the involved parties of the road network. Avoid disclosing their true details to third parties. Identity management of vehicles and passengers.

Decentralization: Protection of VANETs from the failure of centralized infrastructures and design of road systems without centralized control and centralized storage of data and messages. Mechanisms that allow free connection and exchange of data between nodes.

Auditing: Logging of data, messages, and events both locally and remotely with integrity and speed, enabling access to all authorized stakeholders in VANETs.

Sensing Accuracy: Mechanisms to improve the accuracy of vehicle sensors to provide valid and actionable vehicle movement, traffic, and state data.

### 4.2. Social Challenges

Road safety: The road network should provide safety to both vehicle passengers and pedestrians, thus increasing citizens’ trust in the CCAM.

Social Networks: New types of data exchange between vehicle passengers and the rest of the network as well as continuous access to the Internet is a challenge for CCAM and VANET networks.

Resource management: The distribution of resources in processing, storage, and communications is a challenge for the road network and for its nodes (fixed or mobile)

Economy: Techniques and methods to ensure a more cost-efficient use of VANET networks which will help in their green implementation.

### 4.3. Ethical Challenges

Trust management: Improving the reliability and safety of autonomous and interconnected vehicles creates such conditions as to increase people’s trust. Trust is a major challenge of CCAM and VANETs.

Responsibility: Methodologies that will protect the road system from avoiding blaming an accident or incident on the vehicle that causes it.

Accountability: Techniques that record and assign the events to the respective vehicles that cause them without raising issues of the privacy of the users and passengers of the road network.

## 5. Blockchain and Smart Contract Approaches for CCAM Challenges

In order for blockchain and smart contracts mechanisms to be intergraded to existing VANET systems, we initially have to understand the blockchain architecture and the smart contract design. The most common way of integrating them in CCAM is through custom middlewares, which bridge existing transportation systems with the blockchain networks that will be implemented in the vehicles, RSUs, and VANET infrastructure.

The novel approaches that will be analyzed in this section use specific blockchain platforms most often tailored to the applications and technologies used. These platforms are used for the design of the smart contracts, based on application goals and requirements. After that, a middleware layer is created to connect the blockchain with CCAM systems. IoT devices used in VANETs have limited processing power and storage capacity to facilitate blockchain. Edge computing solutions implemented near theses IoT devices and vehicles can reduce the load and satisfy the requirements of blockchain. The implementation of blockchain networks on RSUs acting as gateways can run lightweight smart contracts, filter data, and manage communications with the blockchain network.

**Figure 5:**
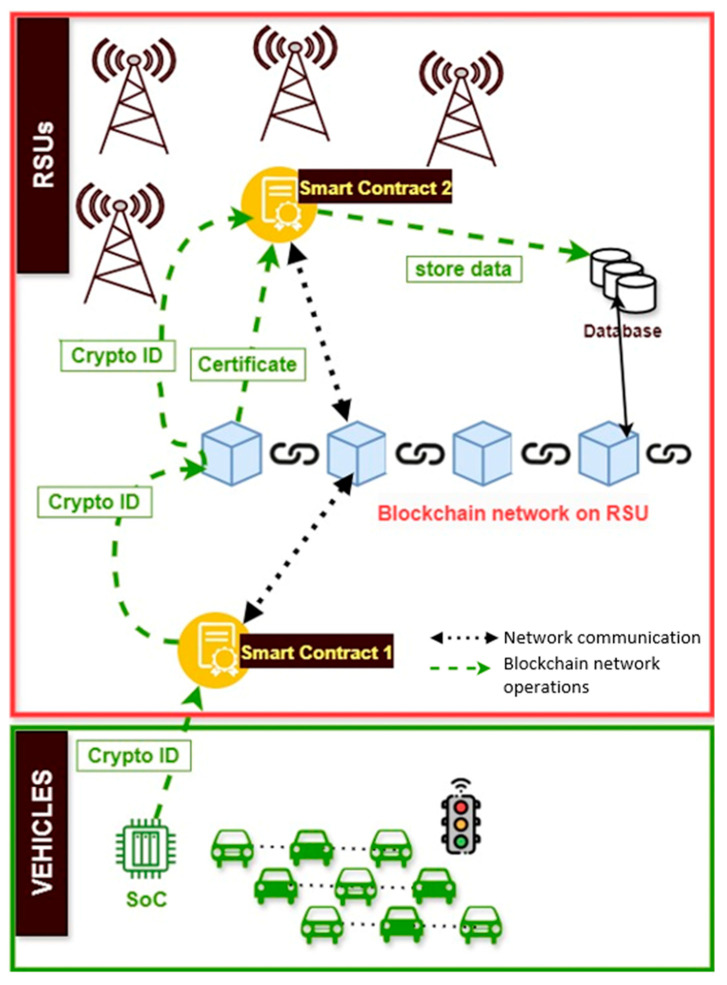
System architecture of blockchain- and smart contracts-enhanced CCAM.

Based on the above, a generic system architecture that integrates smart contracts and blockchain into V2I CCAM systems is illustrated in Figure 5. The blockchain is implemented and maintained in the RSUs due to their greater processing power and storage capacity compared to the edge devices installed at vehicles. Vehicles produce a unique crypto ID and create a public smart contract, which ensures that the data generated by a vehicle and transmitted to the RSU comes from a trusted source, i.e., an authorized connected vehicle. A second but private smart contract is created from the RSU to store and manage the data. In the majority of the related work, smart contracts are coded in the Ethereum Virtual Machine (EVM), which is installed on the RSU. The distance between the RSUs is usually a kilometer or less [42] in order to maintain a high data rate in high traffic. In order to ensure secure communication between the vehicles and RSUs, a unique crypto ID is generated by a system-on-chip (SoC), which is integrated into the vehicles enabling them to connect to the IoV. An example of the mechanism that produce this IDs is presented in [43], which is based on the physical unclonable functions (PUFs), a hardware security primitive characterized by a challenge-response pair (CRP), which exists on the vehicle’s SoCs.

### 5.1. Approaches Targeting Technical Challenges

Connected vehicles, smart infrastructure, and communication networks rely heavily on data exchange between all involved parties in the road network to function efficiently and safely. Protecting this exchange from external or internal attacks and failures is crucial to maintaining the integrity and trustworthiness of CCAM. In order to protect these systems, the key challenges described in Section 4.1 must be addressed with the use of novel methodologies that are based on blockchain and smart contracts technologies. Privacy protection and secure communication are the key technical challenges that must be tackled. Mitigating them without the use of blockchain and smart contracts technologies required a centralized control managing the end-to-end CCAM system architecture (RSU, Vehicles, VANET). But if the central authority is compromised, the entire system’s data can be altered or manipulated. Also, a central trusted entity authority is vulnerable to tampering. These single points of failure are problems for the security and privacy of CCAM systems. The use of blockchain technology and its inherent distributed nature has the power and potential to deliver enhanced encryption, security, and privacy of these systems and make them more reliable with a higher level of security, as made obvious by the methodologies described in the remaining of this section.

The sharing of data between mobile nodes (vehicles) and fixed nodes (RSUs) as well as their storage in distributed storage, with security, reliability, and protection against tampering and identity attacks, is the subject of discussion in [44]. Τo address the security issues in VANETs, the authors propose a framework providing a novel data security sharing and storage system based on blockchain technology with Practical Byzantine Fault Tolerance (PBFT) consensus mechanism. This mechanism addresses the inefficiency of the original Byzantine fault-tolerant consensus algorithm and reduces the complexity of the algorithm, thus solving the problem of data loss and delay in conditions congestion while providing satisfactory error handling in VANET networks. The vehicles with the most frequent contributions offer cooperative intelligence to VANETs and thus have more coins and higher priority to access the data collected locally by the RSUs. This scheme does not rely on a centralized database of trusted third-party entities, so it reduces the cost required for operating and maintaining a centralized database. Also, the use of PBFT makes this approach more advantageous than other solutions in terms of data sharing and storage for vehicles in VANETs. The use of blockchain for storing and transmitting the real identity makes it difficult for an attacker to determine the true identity of the vehicle when a vehicle node is transmitting data. This methodology is suitable for brute force attacks.

The authors of [45] dealt with the geographical scope of VANETs and pointed out that each country maintains its own blockchain network based on Proof of Work (PoW) consensus mechanism, which is limited to the geographical boundaries of the country. The aim of the paper is to describe its security risks, and it specifically focuses on the 51% attack or majority attack, which can undermine the immutability of blockchain technology. The probability is also analyzed under specific parameters such as the number of “good” and “bad” nodes, the propagation time of the messages (delay), as well as the time to calculate the puzzle of the consensus mechanism. The authors define those parameters that ensure message transmission, thus providing a set of guidelines for the design of similar systems. With these guidelines, an attacker, a single entity or group of miners, cannot gain control of more than 50% of the network’s total hashing power. The authors define the above parameters, which makes the application of this mechanism suitable for the prevention of cyberattacks of type 51% attack.

Both security and privacy are issues to be resolved in VANETs. This is highlighted in [46], which is based on the implementation of the Conditional Privacy-Preserving Authentication (CPPA) protocol whose significant limitations and implementation problems are overcome using blockchain technology. Thus, a modified digital signature protocol, ECDSA, is proposed to perform group signature verification by vehicles and RSUs, thereby minimizing the verification cost in VANETs. Certificates of transactions between vehicle OBUs and Certificate Authorities (CAs) are stored in blockchain, while smart contracts are used mainly to display the public key of vehicles with the identity of the transaction in the blockchain. Through the proposed key derivation algorithm that used bitcoin and the adopted digital signature scheme, the blockchain system is capable to resist various cyberattacks: hijacking, man-in-the-middle, 51% attack and unlikability.

Focused on privacy in VANETs, [47] suggests a mechanism for issuing nicknames to vehicles as well as their management is proposed, based on a Public Key Infrastructure (PKI) and permissioned blockchain and smart contract technologies. The authors point out that blockchain technology alone cannot reliably guarantee authentication and nonrepudiation except with the combination of blockchain and PKI. In relation to pre-existing works, this particular methodology provides the possibility of collaboration between several authorities using the above technologies. For this purpose, the architecture is based on three main elements: the blockchain network, which contains nodes (competent authorities, jurisdictions) in which smart contracts are executed, consensus services, authorization and authentication services, RSUs, which are the connecting link between authorities for the certification of nicknames and vehicles, and, finally, the local authorities that are parts of the state and are responsible for managing traffic and enforcing the law on the road network. The authors noted that the performance improvement needs further investigation if we consider that the work does not refer to the parallel and distributed execution of the operations by an RSU. With the registration, authentication, and announcement of vehicles using nicknames based on PKI and permissioned blockchain and smart contract technologies, the proposed solution protects a vehicle from being traced by an attacker.

Decentralization is addressed by methodologies that consider how to transmit the messages from the various events in the road network, without the mediation of a central infrastructure, while considering distributed processing and storage, especially when nodes have resource constraints. Implementing a decentralized road system that can withstand centralized infrastructure failures and facilitate free connection and data exchange between nodes involves several technical and organizational strategies. The use of blockchain for data integrity to create a decentralized ledger that records all transactions between nodes in an immutable and transparent manner and the deployment of smart contracts to automate processes (traffic management, resource allocation, etc.) are a technical solution to cope with the failure of a centralized architecture. Data sharing based on distributed data storage will allow vehicles and RSUs to communicate directly via a mesh network, sharing real-time traffic and road condition data. These techniques provide to the system multiple communication pathways that can be used in the case of failure; also, such a system can ensure continuous, secure, and efficient data exchange between nodes, enhancing the overall safety and efficiency of the transportation network.

In this direction, the authors in [48] propose a Proof-of-Quality-Factor (PoQF) consensus mechanism that runs in four phases and is based on multi-hop message relaying to vehicle mobile nodes for V2V communications. In this mechanism, a critical role is played by the signal-to-interference-noise ratio (SINR), which is the measure for the successful transmission of packets through multi-hop intermediate nodes. The use of polling determines the quality of the signal and thus the delay of the transmission of the packets. In this work, a comparison of the performance of the consensus mechanism with respect to the others is made through simulation, where it is found that it offers moderate performance in terms of security, and low performance in terms of complexity in communications, but, overall, it is more efficient as the nodes increase, making it suitable for environments with large scale. The use of proof-of-stake (PoS) or delegated proof-of-stake (DPoS) consensus mechanisms in blockchain can make a group of validators or delegates collude to manipulate the network (collusion attack). The proposed Proof-of-Quality-Factor (PoQF) mechanism in this work protects the vehicles from collusion attack.

Collaborative location accuracy using Deep Neural Networks (DNN) and blockchain technology and smart contracts is examined in [49]. Aiming to identify the exact position of a vehicle, fixed reference points (landmarks) are used whose exact geographical position is known. With these fixed points, they calculate the errors from the geographical values of the mobile nodes and then train the neural networks according to the corrected data they collect. The corrected positions are stored in a blockchain through which the cooperative vehicles share them. All vehicles share the corrected positions without being revealed to external or internal attackers due to the decentralized and immutable nature of blockchain technology. The architecture of the methodology of [49] includes mobile edge computing nodes (MECs) that have storage space and processing power for blockchain operations and for Delegated Proof of State (DPoS) consensus mechanism but also for performing DNN operations. Both the recording of the location and its sharing between the MECs and the vehicles is done using smart contracts. The protection of the location of the vehicles from an attacker is a security issue. If an attacker finds the location of the vehicle can track the vehicle (tracking attack), making the mobile node vulnerable, the attacker can alter the position of the vehicle (alteration attack). With DNN based on blockchain and smart contracts, mobile nodes are protected from these security attacks.

The methodology of [50] concerns the collection of the data from the vehicles and then sending them through the RSUs to the central servers of the road infrastructure in order to be controlled and used by all involved parties in the road network. For the safe transmission of data, blockchain technology is applied in which the data is encrypted based on the characteristics that concern it. Through the blockchain network, vehicles can access and search for the information they want based on their own characteristics. This auditing procedure is done according to predefined and not dynamic policies. This is also a key drawback of the methodology. The consensus mechanism is based on the Proof-of-Storage mechanism according to which the RSUs with the largest storage contribution are rewarded with storage coins. The authors analyze the performance of the methodology and point out that it is quite efficient compared to previous methodologies, but the time cost for the encryption and decryption operations is similar. This work suggests a solution based on the announcement messages on VANET through blockchain protecting the nodes from alteration and tracking attacks.

The technical aspects of the discussed approaches toward technical challenges of CCAM and a summary of their features are shown in Table 2 and Table 3, respectively.

### 5.2. Approaches Targeting Social Challenges

The methodologies that face the above technical challenges are the basis for addressing more complex challenges that will make CCAM an acceptable solution for people’s transportation in smart cities. The road safety that should be provided by autonomous vehicles for citizens to trust and use will be combined with in-vehicle infotainment systems, thus helping the road system to be able to respond to green mobility and sustainable urban lifestyles. At the same time, the use of resources in financial terms is another challenge of VANET networks.

Road safety and resource management in VANET are main objectives in smart cities, which in their vast majority use centralized networks for monitoring and auditing for detecting incidents and for optimizing the use of resources from the vehicles. The use of centralized real-time monitoring systems can be very slow and can cause delays in incident and threat detection. Also, centralized resource management systems can lead to inefficiencies, as resource allocation decisions might not always be optimal or responsive to real-time demands [16]. Blockchain and smart contracts technologies allow for real-time monitoring and auditing and enable decentralized resource management, where smart contracts automatically allocate resources based on predefined rules and real-time data. This ensures that resources are distributed more efficiently and equitably.

To save resources, especially in storage space during the transmission and storage of the events in a road network with the aim of doing it efficiently and in a short time so as to avoid road accidents, the authors of [42] propose the combination of Transactions Filtering Pattern Matching Scheme (TFPMS) and blockchain technology to ensure data privacy and immutability. The architecture consists of three layers: Vehicular, Edge, and Cloud. All transactions are filtered to reject the wrong ones and save storage space on the central servers located in the cloud. Once the data is filtered, it is stored in the blockchain, and what is deemed important for future use is uploaded to the cloud. With the TFPMS filtering invalid transactions in vehicular networks based on TFPMS consortium blockchain on RSUs for the vehicle communication, this approach protects the vehicles from disclosure their identity (identity attack).

Road safety applications in modern VANET networks are based on the periodic exchange of vehicle status (basic safety messages) via V2X communications over the new 5G New Radio (NR) specification, i.e., 5G NR V2X communications. The purpose of [51] is to improve the performance of New Radio V2X sidelinks (NR-V2X sidelink) over the existing Sensing-Based Semi-Persistent Scheduling (SPS) methodology. The proposed methodology reduces conflicts and improves communication performance by enhancing it by applying blockchain technology with a DPoS consensus mechanism. The vehicles are organized into platoons in which one vehicle is the platoon leader and is responsible for its control, and the other vehicles are the platoon members. This method prevents also the VANETs from collusion and alteration attacks because it uses the platoon scheme that previous described.

The authors of [52] propose a methodology in which electric vehicles (EV) will participate safely but also with the aim of reducing the consumption of electricity in the smart grid network of a smart city. To reduce the risk of the creation of multiple fake identities or nodes from an adversary (sybil attacks) and double spending and to prevent vehicles from operating selfishly when it comes to the use of the energy network, they apply blockchain technologies for attacks and smart contracts for “fairness” in consumption. Also, the use of smart contracts is used to protect customer vehicles and the public electricity network from incorrect and irregular charges.

In [53], a VANET payment system based on blockchain technology is proposed in which the blockchain network is maintained in RSUs and the vehicles generate the transaction content. In the proposed model, vehicles relay on the lower level. On the higher level, there is the blockchain layer. Trading between one vehicle and one RSU is called V-R transaction, while trading between one vehicle and multiple RSUs is called V-Rs transaction. These two types of transactions are applied, respectively, in the scenarios park toll management system and electronic toll collection system. Although this particular methodology is a simple approach without considering how blocks are verified, it safeguards the user accounts of the mobile vehicles from unauthorized access, misuse, and other security threats because of the use of smart contracts between RSU and vehicles.

Electric cars’ range and ways to set up an effective charging infrastructure is the main subject of [54]. The lack of charging stations can be addressed by exchanging energy from neighboring vehicles that have available energy. To design and implement this idea, we should consider parameters such as the availability of charging stations, the length of time for charge, the cost, and the reliability of the service. The problem with this idea is the effective supplier selection along with the security and the uninterrupted exchange between buyer and seller. This methodology provides a mechanism for a novel VANET-based multi-criteria supplier selection based on the Named Data Networking (NDN) framework and a blockchain technology to guarantee security and uninterrupted exchange. The basic features of the model are trust enforcement, secure authentication of trading entities, and supplier selection, making this approach suitable for protection from 51% attack type between energy supplier and vehicle. The suggested approach compared to previous works shortens the time spent looking for suppliers and reduces costs for purchasers.

To overcome problems of security, computational capabilities, and especially scalability in the Social Internet of Vehicles (Social IoV), a methodology is proposed in [55] based on a two-dimensional blockchain and a dynamic consensus mechanism that implements the use of checkpoint blocks for better use of vehicle resources and mobile points at the “edge” of the network. The architecture of the work consists of the vehicles, the RSUs that act as miners but also are responsible for the registration of the vehicles, the Server/Miner, which provides the blockchain network, the edge modules, which are responsible for the protection from “overflow” of vehicle data but also for the management and allocation of resources. The consensus mechanism is dynamic PoW (dPoW), which consists of four levels of difficulty that are applied depending on the rate of incoming traffic to SIoV data and that balance security with performance. The proposed methodology is evaluated according to the parameters scalability, security, privacy, and latency. With the two-dimensional blockchain and the mining on the RSU, this methodology prevents the network from unauthorized takeover or control of a system, account, or communication channel by an attacker (hijacking attack) and also from different kinds of attacks such as tampering, replaying, and packet injection.

The technical aspects of the discussed approaches toward social challenges of CCAM and a summary of their features are shown in Table 4 and Table 5, respectively.

### 5.3. Approaches Targeting Ethical Challenges

If the above challenges are addressed, they will offer a more mature and reliable connected and autonomous driving, thus increasing the trust of citizens as well as the degree of penetration of CAVs in daily commuting. Safe driving and economy in time and cost as found in the previous section are key parameters for this penetration. This study highlights trust as an important ethical challenge for the CCAM. At the same time, the accountability of an accident should be based on parameters such that the result is not disputed. The person responsible for the accident should not be able to deny the incident he/she caused. But these systems, as is also the case with vehicular networks, operate in an open-access environment that gets more exposed to attackers. In addition, vehicular network users dread infringement of their protection and privacy of their data. These issues do not improve the trust, the accountability, and the transparency of CCAM. However, traditional trust and transparency mechanisms are based in a central authority or a limited number of trusted entities’ decisions. These mechanisms and their decisions are often opaque with limited transparency, making it harder to trace the origin of data or actions, leading to potential trust issues. Blockchain provides a transparent and immutable record of all resource allocation decisions. Every action is traceable to a specific entity or smart contract, and every transaction is recorded on the blockchain, allowing all stakeholders to see and verify this transaction [20].

In this direction, the authors of the paper [56] propose a framework in which they combine blockchain and named data networking technologies with awareness of privacy and security in V2X communications and that they call Secure-V2X. This particular methodology does not use the private information of the parties involved (drivers, passengers, pedestrians, etc.) but non-private information such as the license plate number. An important addition to the methodology is that the maintenance and preservation of the blockchain network is not based on RSUs or other fixed infrastructures but on vehicles that are organized in clusters. To achieve consensus, multiple head vehicles participate in the process, making the methodology suitable for protection against privacy attacks as well as DoS. The aim of the work is to assign responsibility to the vehicles without revealing their identity.

In order to maintain immutability but also make it possible to hold vehicles accountable when sharing data collected through VANETs, the authors of [57] proposed combining blockchain technologies and smart contracts in parallel with the new features introduced by 5G communication networks and the media management of Software-Defined Networks (SDNs) technology. Authorized users store the data in the blockchain network, while large files such as video files are stored in an InterPlanetary File System (IPFS). Those users who produce the message data are called owners, while the rest of the users are the consumers and who search for the messages. There is also the Trusted Authority (TA), which is a trusted off-chain third party to create the system parameters, distribute keys, and deploy smart contracts. The above model also involves the blockchain network to which both owners and consumer users have access. The network architecture is based on SDN technology to reduce delays in uploading and downloading data through 5G communications. The registration of messages by user-owners is based on keywords, which words are searched for by user-consumers by confirming them using smart contracts. An adversary to get a keyword by keyword guessing attack is negligible (alteration attack). Therefore, the proposed approach can achieve the searchability with privacy protection.

In the work of [58], presented is a reputation evaluation model based on the logistic regression model by quantifying the behavior records of the distributed authentication entities. The methodology is a Hierarchical Certificate Service Chain (HCSC) based on reputation by introducing Master Authorities (MAs), CAs, and Roadside Unit Authorities (RSAs) in the blockchain network to monitor Authentication Entities (AEs) for providing reliable and transparent certifications. The suggested model is based on the blockchain architecture with four layers: data, network, consensus, and application and is efficient for the management of distributed authentication entities. The performance of certificate service is suitable for node authentication in VANETs. The certificate service models of the methodology are divided into two categories, roughly: the traditional distributed certificate service model and the blockchain-based model. With these models, the authentication entities are protected by various internal and external attacks, such as unauthorized interception to communications and data transmissions between nodes (eavesdropping attack), interception of legitimate data transmission and retransmissions (replay attacks), and man-in-the-middle attacks.

The methodology of [59] is a combination of blockchain, SDN (software-defined networking), and fog computing technologies to effectively manage and control the network in VANETs. The above technologies use 5G communication technology and fog computing technology to avoid frequent base changes (handovers) from vehicles, while the blockchain layer is included in the control plane of SDN. Also, the implemented consensus mechanism is PBFT and is used in order to ensure consistency between many involved entities in the model. RSUs act as miners, while the leader is elected from among themselves to create the blocks of the chain. There is a small group of default nodes that participate in the voting process to verify a block before reaching consensus. The methodology is based on immutable and distributed blockchain features to support the trust of messages, which are evaluated and scored by giving them a reputation score that is registered in the blockchain. With the SDN being centrally controlled, it becomes vulnerable to denial-of-service (DOS) attacks and also suffers from a single point of failure [58], such that a failed response by the SDN controller limits connectivity in the 5G network [58]. The suggested methodology uses a blockchain layer in the control plane of SDN and protects the network from DDoS attacks.

The authors of the work [60] analyze the importance of responsibility and the fairness of VANET vehicles and propose a methodology based on the calculation of the reputation value of a node that is stored in a distributed network and while the remaining nodes searches through smart contract technology. Each vehicle not only has the right to investigate the reputation of a node, but also contributes to its evaluation process, having a clear view of what is happening in the evaluation process. The storage of the messages of the transmitted events is not done in a blockchain network but in the distributed IPFS network, which costs less and has greater potential in storage space. Through smart contracts, it is ensured that only the authorized party modifies the content, shares and updates the blockchain in the form of an IPFS hash of the hash ID. The above methodology provides decentralization, transparency, and immutability to peer nodes, which leads to consistency of the reputation value of each user. To avoid badmouthing, a decentralized blockchain network with a quick consensus algorithm is deployed for reputation evaluation. Before a node adds the reputation score corresponding to a node, it should be verified and then added. With this colluded badmouthing or commendation should be avoided (collusion attack).

The work of [43] concerns the design of a solution for trust management and for secure data transmission between vehicles and RSUs using the physical unclonable function performed by the embedded chips (SoCs) located at the vehicles participating in the vehicular network along with blockchain technology. The use of PUFs gives each smart vehicle a unique cryptographic fingerprint that is used to determine the origin of the data. The network that supports the above solution is called DrivMan, and for the communication and confirmation of the data transmission it uses two smart contracts, a public one between the network and the RSUs and a private one between the RSU and the vehicle. By using the above technologies and using a PKI, the authors propose the DrivMan methodology to facilitate trust management, data provenance, and privacy. The authors assume that RSUs and the blockchain network do not have resource constraints, unlike the vehicle, which does. Also, vehicles have SoCs with PUFs, and any attempt to tamper with or remove PUFs will render communication useless (alteration and identity attack). The goal of the attacker is to correlate these vehicle data to reveal identities, patterns, or relationships (unlikability attack) that were intended to be kept private is prevented with the use of private smart contract between the RSU and the vehicle and with the PUF’s cID.

The technical aspects of the discussed approaches toward ethical challenges of CCAM and a summary of their features are shown in Table 6 and Table 7, respectively.

The primary purpose of all the previously presented methodologies is to deal with some attack or threat from malicious or untrusted nodes. According to [21], there are many and different types of attacks and threats, which must be addressed in order to make CCAM and CAVs suitable and reliable for daily safe use in transport. The types of attacks/threats that the blockchain and smart contract methodologies studied in this paper address are summarized in Table 8.

## 6. Discussion and Future Directions

VANETs, especially CCAM, require high reliability and very low or zero delays. It is also particularly important to use computing and communication resources in a distributed and decentralized manner. For this purpose, blockchain technologies and smart contracts comprise an important application field of VANET networks. The analysis of the different methodologies described in the previous section reveals that the vast majority of studies addresses technical challenges, and some of them go a step further and tackle higher-level challenges such as social and ethical. Technical challenges include fundamental requirements such as data transmission security, privacy protection, accurate and reliable recording of events and messages, improved the data output by vehicle computing systems and sensors, and distributed and decentralized operation of VANETs [15]. Social and ethical challenges have significant value and effect on daily lives of people who will use CCAM. Starting from addressing issues related to road safety, the economy, and environmental protection, we end up with issues of accountability and responsibility and, finally, of trust and safety [13].

What we can observe is that not all the methodologies examined in our study face CCAM challenges using a combination of blockchain and smart contracts. Specifically, about a third (35%) of them rely on smart contracts over blockchain mainly based on the Ethereum platform. There is also high variation in the implementation of consent mechanisms. Most of the works apply the classic mechanisms of blockchain networks: Proof-of-Work, Proof-of-Stake, Delegated Proof-of-Stake, Proof-of-Authority, and Practical Byzantine Fault Tolerance. However, several of them implement improved or enhanced mechanisms in terms of performance and communication load. Examples of these mechanisms are Enhanced Delegated Proof-of-Stake, Dynamic Proof-of-Work, Crash Fault Tolerance, and Directed Acyclic Graph. Fewer works employ specialized or new mechanisms that perform better for specific application needs, such as Proof-of-Storage, Proof-of-Quality-Factor, and Adaptive Delegate Consensus algorithm.

Due to the requirements in processing power, storage space, and communication range, the functions of the blockchain network, and the corresponding consensus mechanisms, most methodologies apply the block mining process and the consensus mechanism either to the RSU nodes or to the servers located in central infrastructures or infrastructures at the edges of the network (edge servers) [21]. However, the use of central cloud-based infrastructures for mining and block creation is not in the direction of decentralized and distributed VANETs, which is a critical factor for effective large-scale deployment of CCAM in smart cities. On the other hand, the important role of RSUs is mentioned in all the works since they are the means of communication and interconnection with the central network and the Internet of vehicles.

Although most of the proposed methodologies were evaluated through simulations, there are a few methodologies that use real-time data from smart cities. The methodology proposed in [55] used real-time data from the region of Little India in Singapore with the following parameters: a route length of 1471.14 m and 100 nodes with left-hand driving orientation, moving with a uniform speed of 30 m/s and no pause time. The framework of this works allows to scale and accommodate the ever-increasing transaction traffic and provides rich computing resources for the vehicles to extend their resource limitations by offloading their tasks to the edge modules. A real-time environment is also used in [57], where vehicular trace generated from the OpenStreetMap is collected from the traffic flow on Taipei roads. Although this methodology reduces message transmission time and network load over 5G VANET using SDN technology, it has a low performance in a real smart city. Another example of blockchain application in a smart city is the Shared Mobility Intelligence system, which uses permissioned blockchains. This system records and shares the routes of share vehicles that travel in four different routes of Kerala, India [61]. This work studies four different cases while allotting seats in SVs for travelers/residents of smart cities. Because most of the proposed methodologies were evaluated through simulations, this raises concerns about the performance of the methodologies in real environments and in real-world implementations of the road ecosystem.

Compared to other approaches, the cost, efficiency, and ease of implementation the methodologies based on blockchain depends on different parameters and criteria. For example, schemes that do not rely on a centralized database of trusted third-party entities reduce the cost required for maintaining a centralized database. Also, the use of PBFT instead of other traditional consensus mechanisms make more advantageous the methodologies than other solutions in terms of data sharing and storage for vehicles in VANETs. The ease of implementation depends strongly to the selected consensus mechanism. The author of [48] showed that a completely distributed P2P blockchain in VANETs with the least possible reliance on RSU and the infrastructure is not possible to be implemented with PoW, but an RSU-dependent network will be a costly solution. A joint PoW and PoS consensus managed by RSUs is proposed for easier implementation and better performance. The approach employed in [54] shortens the time spent looking for suppliers and reduces costs for purchasers compared to previous works. The methodology in [53] reduces costs because the necessary costs (such as human cost, infrastructure construction cost) will be generated, which is important to build a healthy ecology. On the other hand, the cost depends on the increase of vehicle density and speed, where the signaling cost is rising [53]. While blockchain can reduce costs in a long time period, the initial investment in implementation, infrastructure, and training can be significant. Organizations need to consider the cost-–benefit analysis over time.

Our literature review revealed various potential future directions for blockchain and smart contracts in CCAM, as summarized in Table 9. It is a requirement of CCAM users to create well-structured trust models to create an environment of trust and acceptance by smart city citizens toward smart road networks [20]. These models should be based on entities, on data exchanged, and on context constraints considering different properties, metrics, and parameters. As described in Section 5, many of the tasks address the various challenges with moderate or low performance and with low QoS indices. To increase the QoS indicators, common properties and characteristics should be defined on which future research and studies of VANETs and CCAMs will be based [62]. Examples of these properties or characteristics are reliable and accurate data transmission, communication cost, user privacy, and data security. On the other hand, once the common characteristics are defined, and, in accordance with them, the new methodologies are applied, their results should be evaluated according to specific evaluation parameters. In other words, specific but also common evaluation parameters should be defined for all parties involved in the research process and study. Security evaluation parameters and metrics refer to delays during data sharing by vehicles, where the main computational delay stems from verifying messages and the transmission confirmation time between nodes. The cost per transaction measures the cost incurred for processing a single transaction on the blockchain and also the communication costs are evaluation metrics for social challenges. Another important metric concerns scalability, which can be measured in terms of transaction throughput and latency changes as the network size increases. Finally, in the case of trust, reputation-based metrics must be defined based on the recommendations and opinions given about a specific node within the network.

The characteristics of blockchain technology are a building block to protect against different kinds of attacks with the goal of securing communications, privacy, and failure of systems that manage VANET networks. However, in each of the methodologies studied in the work, it was found that they do not cover a wide range of different types of attacks [45]. Perhaps the combination of several methodologies can provide protection against a set of attacks, proposing a framework that will fully protect VANETs and autonomous and connected vehicles from attacks. The resilience of CCAM to such attacks is critical for the safety of people and especially for services provided such as emergency transport in the event of accidents or disasters (ambulances, fire and police vehicles). The coordination and communication of various VANET components need to ensure that they will be protected from system and network fault tolerance, especially in the implementation of blockchain and smart contract technologies. Although the decentralized nature of blockchain reduces single points of failure and enhances the system’s overall robustness and fault tolerance protection, future studies have to include new methods for protection of fault tolerance: redundancy in IoT hardware components with different types of sensors, smart contract automated recovery procedures on specific failures, load balancing methods for preventing from system overload, and resources overuse.

The applied methodologies should also consider environmental parameters primarily aiming to decrease the consumption of energy and resources [63]. The performance of the methodologies should be evaluated in the direction of reducing the load and delays in the various complex processes and calculations, thus creating frameworks with a small energy footprint and less burden on the environment. In this way, the CCAM will be a key structural component of smart and green mobility.

Moreover, various methodologies related to trust management, accountability, and road safety, were based on the application of techniques and systems that evaluate, reward or not, users but also create the parameter of reputation in profile of the vehicle or the user [20]. These techniques rate a vehicle and create a reputation for that vehicle, based on its behavior in the road network and its contribution to traffic data sharing as well as to the transmission and confirmation of a road incident. However, each of the above methodologies examines different entities that participate in the road ecosystem: some concern the behavior of driver/passengers, others the behavior of vehicles, and others the reliability of fixed nodes such as RSUs. Therefore, to improve reliability and trust, systems should be designed that involve all the entities: fixed and mobile hubs, drivers and passengers, but also pedestrians or users of bicycles, skates, and public transport. These systems will include the creation of an overall profile that will have as parameters: the reputation of each entity, the degree of contribution, and the overall score in the road ecosystem of the smart city.

An important technology in VANET networks is AI, which is already applied to some of the methodologies we reviewed, with the aim of better data transmission, evaluation, and decision making. AI can help developers, decision-makers, regulators, end-users, and CCAM service providers to use global intelligence of the entire IoV ecosystem [64]. For example, AI can optimize routes based on factors like traffic congestion, road conditions, and destination, ensuring efficient and safe navigation vehicle path planning. Also, AI algorithms can control vehicle movement and operations (steering, acceleration, and braking), enabling autonomous operation in various driving scenarios. Optimizing these models is a major challenge of the methodologies. To complete this optimization and make the CCAM trustworthy and reliable, Federated Learning (FL) needs to be implemented alongside AI [65]. Collaborative learning by collecting knowledge from different devices or entities will improve the overall intelligence of the system, thus having a significant impact on building trust between human and autonomous vehicle as well as the road safety of CCAM. For example, sharing maps can improve the accuracy and completeness of the map. Vehicles can train in real time, making better decisions, reducing latency, and improving responsiveness [66]. Collected knowledge from vehicles can help mobile nodes with tasks like object detection and traffic prediction. Each vehicle contributes its data to the global model, improving its accuracy over time. Nevertheless, the combination of blockchain technology with FL and AI in environments without centralized management but in a distributed network of cooperative intelligence is the subject of further study and investigation.

Along with the previous technologies, other emerging technologies should be implemented, such as fog and edge computing, SDN, and Network Functions Virtualization (NFV) [67,68]. Their implementation should be based on future research and methodologies combined with blockchain and IoV technologies to extend the original VANET networks to V2X communications. The above technologies will help to provide better QoS indicators and architectures that will ensure dynamic, reliable, and secure trust management as well as performance of responsibilities to the parties involved in smart mobility while at the same time using blockchain, data will remain immutable, durable, and traceable. Cloud, SDN, and NFV technologies create conditions of dynamism, scalability, better network management, and resource sharing [69]. However, architectures and methodologies based on cloud and SDN services need further investigation due to both the delay of data transmission between nodes and infrastructures, as well as security and privacy issues that arise.

In many of the methodologies we studied, performance issues of the functions required by blockchain and smart contract technologies were observed, especially during the creation of the blocks and during the execution of consensus mechanisms. The performance issues concerned both the delay of the above processes, the energy consumption, and the communication load they create. Autonomous and interconnected vehicles are the most important part of CCAM. The latter provides citizens with many IoT and IoV applications based on blockchain technology. Each of these applications has different characteristics and requirements: a road emergency system has different delay requirement from a parking space management system. Another example is the vehicle’s collision avoidance mechanism, which requires zero delays and reliable, error-free data transmission [70]. Therefore, studies and research should carefully consider the diverse requirements and focus on the design of more efficient frameworks utilizing blockchain for heterogeneous applications, in order to apply the optimal blockchain and smart contract technologies and improve their performance.

Conventional PoW-based blockchain methodologies in IoV suffers from poor resource pool and computational complexity [55]. Also, their high energy consumption and slow transaction processing creates difficulties of the application of blockchain and smart contracts in large-scale environments. The latest and novel mechanisms like PoS, DPoS, and BFT variants offer faster and more scalable alternatives, making them more suitable for large transportation networks. To address scalability obstacles, new methodologies must be studied in different directions. Parallel blockchain must be used, one for the road network and the other for the transport network. Also, different blockchain solution and platforms must operate as one system in order to communicate and transact with each other. Finally, the methodologies that are studied in this work must be first tested in smaller, controlled environments before being scaled up to cover larger networks. Scaling blockchain solutions in complex transportation networks is a challenging task that needs advanced solutions in technology, management, and infrastructure.

The overall performance of the network due to the exchange of a large amount of data before the blockchain consensus is reached is affected by the large network load of maintaining the blockchain network. The decentralized nature of the latter and the consensus mechanism results in an increase in network load and delays, which causes a significant problem, especially in cases where the range of communications is limited. So, the above should be considered when implementing blockchain in VANETs as it is necessary to consider the balance between decentralization and network load.

This study presented a rich variety of methodologies that deal with 12 specific challenges. Implementation of these methodologies, particularly in real-world settings, could potentially reveal new challenges. The absence of widely accepted standards for blockchain and smart contracts in the CCAM as well as the inability of different blockchain platforms to work together seamlessly are challenges not covered in this work. Apart from these interoperability and standardization challenges, future works must focus on methodologies that deal with regulatory and legal challenges. The legal status of blockchain and smart contracts varies by jurisdiction, leading to potential conflicts with existing laws.

### 6.1. Use of Distributed Hash Tables in CCAM

A special reference is made to Distributed Hash Tables (DHT), which provide a distributed and scalable way to store and retrieve key-value pairs across a network of nodes. New approaches using DHT can enable the creation of large-scale, decentralized systems for storing and retrieving data without relying on a central authority. The need to deploy decentralized CCAM systems relying more and more on edge computing architectures leads to the use of peer-to-peer (P2P) solutions, which decrease the delay, are more tolerant to failures, and faster than other options. Toward this direction, in a non-blockchain context, IPFSs are frequently used to support P2P interactions between IoT sensors, RSUs, vehicles, and other actors, where DHTs can play a pivotal role in content querying, retrieval, and sharing [71]. DHTs enable peers to locate the required content within the network simply using its cryptographic hash, guaranteeing content integrity and security and a more efficient operation of the VANET through peer selection mechanisms based on network-related criteria.

DHTs can be implemented in various ways in tandem with blockchain technologies. For instance, data exchanged between vehicles and RSUs can be stored in a DHT through an IPFS. The DHT produces hashes that are then maintained in the blockchain network [72]. Optimized use of resources can be achieved by a PoA consensus mechanism and a cache server located at the edge of vehicular network. A three-layer approach is proposed in [73] with a CA at the first layer, an IPFS integrated into the RSUs storing DHTs at the second layer, and the hashes stored in the blockchain network at the third layer. The use of DHTs and the storage of data hashes in the blockchain network instead of storing the data themselves may lead up to a 15–18% reduction in required computational resources and a 80–85% reduction in memory overhead. Moreover, DHTs in conjunction with blockchain prevent tampering and improve validity of the transmitted data.

There are various CCAM applications where DHTs provide a more secure, reliable, and transparent data sharing environment, while enabling distributed data storage such as in the Internet of Electric Vehicles (IoEV) and traffic monitoring and management by drones. In the former, DHTs can be utilized with blockchain to protect data and energy trade, laying the foundation for an efficient, scalable, and reliable EV energy management framework [74]. Security and integrity of flight data in a blockchain solution for handling a traffic management system based on unmanned aerial vehicles can be reinforced with the use of DHTs [75].

### 6.2. The Role of 5G Communications in CCAM

Tightly connected to the challenges presented earlier—particularly those related to performance, decentralization, distributed resource management, and scalability—is the emergence of fifth-generation (5G) mobile communication technology, which is expected to greatly contribute to tackling them. 5G is expected to pave the ground for innovative mobility schemes and services that require very low latency communications, reshaping transportation and affecting all types of connectivity included in CCAM [76]. It can handle more complex and denser network configurations by utilizing a wider connectivity range. This is very beneficial for V2I interactions employing a huge number of simultaneously communicating connected devices, such as in smart cities, where apart from vehicles, charging stations, traffic lights, traffic management sensing devices, smart road signs, and other components participate in service delivery [77].

Additionally, 5G integrates all existing communication technologies through techniques such as network slicing, which allows for the allocation of different network slices to different applications based on their specific network requirements [78]. For instance, applications targeting improved safety such as cooperative localization require reliable and with low latency data exchange, while on-board entertainment applications demand increased transmission capacity. 5G-enhanced communication standards for V2X connectivity are been developed to support advanced driving applications. 5G-enabled CAVs are able to accurately share their status in terms of location, orientation, and speed for various V2I-related applications such as obstacle warning, navigation, and path planning [79].

5G thus offers the ability to make real-time decisions toward improved safety and reduced accidents. When blockchain technology is combined with 5G for CAVs, it can guarantee that only authorized vehicles gain access to the network. Additionally, blockchain safeguards the integrity of the data stored within it, ensuring it cannot be altered. Combining beyond 5G with blockchain guarantees a secure and distributed method of delivering services [80]. It can be summarized that 5G-enabled CCAM systems will enable enhanced communication between vehicles and vehicles, as well as between vehicles and infrastructures delivering on the promise of increased speed, reliability, and scalability potential. This will effectively improve safety, flexibility, smartness, and efficiency of CCAM networks and applications.

## 7. Conclusions

This study presented the basic challenges of cooperative, connected, and automated mobility, grouping them in three categories: technical, social, and ethical. Contrary to previous related surveys, we explore solutions for addressing all three categories of challenges in vehicular networks. First, we identified and described 12 major challenges, and then we analyzed the most contemporary methodologies and techniques that use blockchain and smart contracts to deal with each challenge. Additionally, we took a deep dive into the technical features, advantages, disadvantages, and impact of each methodology providing a comparative analysis, and we identified different types of attacks and threats they deal with. It was observed that the majority of blockchain approaches utilize classic mechanisms, such as PoW, PoS, DPoS, PoA, and PBFT, while the majority of smart contracts employed in CCAM systems rely on Ethereum. Most of the solutions described in the literature are validated through simulations, with only a few studies performing real-world experiments with real-time data. Decentralization is not yet fully achieved since several approaches use cloud infrastructures; however, there is a pervasive tendency to move computational and network operation at the edge with RSUs playing a fundamental role as devices in the fog. We critically discuss our findings and attempt to delineate the future directions and technological trends intertwined with CCAM, placing a special emphasis on 5G and DHTs. What can lie concluded is that there is still a lot of work to be done to overcome the challenges deriving from the highly distributed, open, and dynamic transport ecosystem, so that blockchain and smart contracts can be used efficiently in large-scale CCAM systems.

## Figures and Tables

**Table 1:** Comparison of relevant surveys.

Survey	Technical Challenges	Social Challenges	Ethical Challenges	Blockchain Approaches
[12,15,16]	✓			
[13]	✓			✓
[14,17,18]		✓	✓	
[19]			✓	
[20]	✓		✓	
This work	✓	✓	✓	✓

**Table 2:** Technical aspects of blockchain and smart contracts’ methodologies for technical CCAM challenges.

Ref	Challenge	Blockchain/ Smart Contract	Consensus Mechanism	Techniques/Tools	Performance of the Methodology
[44]	Security	Blockchain	PoW and PBFT	-	Better performance in computation and transmission times as the number of verification signatures increases, compared to existing solutions (IBV, SPRING, IBCPPA, and EAAP)
[45]	Security	Blockchain	PoW	-	It implements the BIP325 key extraction algorithm to avoid preloading keys and burdening OBUs with storage consumption. The performance of the technique is not affected by the average speed as far as packet loss is concerned
[46]	Security	Permissionless Blockchain and Smart Contracts (Ethereum)	PoW & PoS	-	The methodology is efficient for small delay time in the transmission of messages from the group of “good” nodes
[47]	Privacy	Blockchain	PoW	Distributed Cloud Servers	It achieves fewer cycles (steps) in communication compared to pre-existing methodologies
[48]	Decentralization	Blockchain	PoQF	Game Theory, VEC network	Compared to the rest of the consensus mechanisms studied, it has less loss when validating events, but this has the impact of the longest delay in message transmission
[49]	Sensing Accuracy	Permissioned Blockchain and Smart Contracts	DPoS	DNNs	Position correction compared to other methodologies is more effective when we have many errors from the sensors
[50]	Audit	Blockchain	PoS	-	Moderate transmission speed performance—High security

**Table 3:** Features of blockchain and smart contract methodologies for technical CCAM challenges.

Ref	Challenge	Technology	Advantages	Disadvantages	Impact
[44]	Security	Blockchain—PoW and PBFT	Protection against attacks on centralized systems and malicious RSUs Protection against brute force attacks based on asymmetric encryption and signature verification techniques.	With a small number of involved RSUs in the network, there is an increased possibility of malicious tampering; thus, the system is unstable.	Data collected from the RSUs of a VANET are protected by brute force attacks through distributed data storage based on blockchain technology and from the exchange of data between RSU and vehicles using smart contracts, making the network security more suitable for CCAM environments
[45]	Security	Blockchain—PoW	It defines those parameters that ensure the secure transmission of messages in VANETs located in a limited geographical area.	Even with a small percentage of malicious nodes, if the delay time of messages from malicious nodes is less than “good” nodes, the 51% attack is quite possible.	The blockchain network is protected against malicious nodes, even if these “bad” nodes form a group of miners, due to the guidelines and parameters that are defined.
[46]	Security	Permissionless Blockchain and Smart Contracts (Ethereum)—PoW & PoS	Provides security against various types of attacks, such as hijacking, 51% resistance to attacks, DDoS, man-in-the-middle	The average packet delay of the data is affected by changes in the average speed of the vehicles.	Presentation of the new CPPA protocol for establishing secure communications in VANET networks, utilizing blockchain technology.
[47]	Privacy	Blockchain—PoW	Efficient methodology reducing the dependency on the CA and the burden on vehicle authentication	It is not a purely decentralized solution because it is based on a relatively small number of servers in the cloud.	Mechanism for adding extra protection against transmission of false messages in vehicle transactions in V2V and V2I communications that guarantee authentication and nonrepudiation in combination of blockchain and PKI.
[48]	Decentralization	Blockchain—PoQF	Reliable mechanism in case of knotting. Fewer validation losses than other consensus mechanisms.	The voting mechanism increases latency. It is impervious to 51% majority attacks.	A novel consensus mechanism for VANET, differing from existing systems, where node votes on road events and accidents are used. Security protection against collusion attacks is provided through a blockchain-based PoQFconsensus mechanism.
[49]	Sensing Accuracy	Permissioned Blockchain and Smart Contracts—DPoS	Improvement in position accuracy is possible even when access to reference points is interrupted	It does not consider random errors in positioning	Integrating DNNs with blockchain and smart contracts to enhance and share location accuracy, eliminating the need for centralized management by utilizing mobile edge computing nodes and vehicles.
[50]	Audit	Blockchain—PoS	It reduces the need for processing power in vehicles	Stored feature policies are not dynamic and do not change.	Definition of quality characteristics stored in the blockchain network for evaluating and recording vehicle announcements. The blockchain safeguards nodes against alteration and tracking attacks.

**Table 4:** Technical aspects of blockchain and smart contracts’ methodologies for social CCAM challenges.

Ref	Challenge	Blockchain/ Smart Contract	Consensus Mechanism	Techniques/Tools	Performance of the Methodology
[42]	Road Safety	Blockchain	PoW	Distributed Cloud Servers	Low efficiency: Linear increase in both storage space and operating costs in line with the increase in vehicles
[51]	Road Safety	Blockchain	DPoS	5G NR V2X	High performance compared to the SPS technique in terms of collision probability and delay.
[52]	Resource Management	Consortium Blockchain and Smart Contracts	Proof of Authority (PoA)	Smart Grid	Moderate energy saving performance compared to existing solutions.
[53]	Financial	Blockchain	PoW	-	High performance in relation to the time needed to search for a location but also the reduction of congestion and costs
[54]	Financial	Blockchain	PoW	NDN, Vehicular Sensor Networks	Moderate performance relative to pre-existing works. Effectiveness: collection reporting, fake identical rate, and time for trade
[55]	Social Networking	Permissioned Blockchain	dPoW	-	High: Compared to existing methodologies, this one performs better on a large increase in social network data and offloads vehicles from resource consumption

**Table 5:** Features of blockchain and smart contract methodologies for social CCAM challenges.

Ref	Challenge	Technology	Advantages	Disadvantages	Impact
[42]	Road Safety	Blockchain—PoW	It reduces the need for processing power in vehicles	Stored feature policies are not dynamic and do not change.	Improving the performance of communications in VANET road safety applications with the help of blockchain technology
[51]	Road Safety	Blockchain—DpoS	Efficient technique in a large and dense number of vehicles	-	The protection against false road events, filtered and stored in the blockchain network using 5G communication, is enhanced in terms of performance and safeguarded against alteration attacks.
[52]	Resource Management	Consortium Blockchain and Smart Contracts—PoA	Elimination of cheaters, complete supplier coverage with short time of searching and reduces costs for purchasers	There is no integration with IoV Infrastructure	Enhance existing energy stations to save energy and protect against Sybil attacks by utilizing blockchain and smart contracts.
[53]	Financial	Blockchain—PoW	The communication load increases linearly in relation to the number of vehicles and not exponentially as it happens in pre-existing techniques.	Data transmission performance decreases for vehicles that are further away from other RSUs.	Application of blockchain on a higher level of VANET architecture for the implementation of an electronic payment system for the vehicles
[54]	Financial	Blockchain—PoW	Fast transaction transfer	The authentication mechanism and communications architecture are not described	Energy exchange in VANET requires a secure and reliable supplier, as well as trusted neighboring vehicles. This methodology presents an effective supplier selection mechanism with secure buyer/seller exchanges for Smart EV charging, aimed at reducing anxiety in VANETs using blockchain technology.
[55]	Social Networking	Permissioned Blockchain—dPoW	Low delays in V2I communications	Cloud servers pose a problem as far as the distributed feature of the methodology is concerned	Integrating and managing social network data exchange between IoV in existing VANETs using a dPoW mechanism within the blockchain network.

**Table 6:** Technical aspects of blockchain and smart contracts’ methodologies for ethical CCAM challenges.

Ref	Challenge	Blockchain/ Smart Contract	Consensus Mechanism	Techniques/Tools	Performance of the Methodology
[56]	Accountability	Blockchain	PoW	NDN	Moderate performance in the communication load due to the handling of a large amount of data by the vehicles but also due to the different key pairs used in the technologies based on
[57]	Accountability	Permissioned Blockchain and Smart Contracts	PoW	IPFS, SDNs	Low performance
[58]	Accountability	Blockchain and Smart Contracts	DPoS, PoW		Better for large concurrent authentication requests than a large number of requests
[59]	Responsibility	Blockchain	PBFT	SDN, Fog computing	Moderate performance in terms of communication load
[60]	Responsibility	Permissioned Blockchain and Smart Contracts	PoA	IPFS	Low performance as the number of malicious nodes increases, compared to the methodology without smart contracts
[43]	Trust management	Blockchain and Smart Contracts	PoW and PoS	PUFs	Works effectively against data tampering and identity disclosure attacks

**Table 7:** Features of blockchain and smart contract methodologies for ethical CCAM challenges.

Ref	Challenge	Technology	Advantages	Disadvantages	Impact
[56]	Accountability	Blockchain—PoW	The identity of the parties involved in the road network is not disclosed. It is an appropriate methodology to protect against identity disclosure and non-attribution attacks.	It has no filtering techniques for the data generated by the vehicle. Using different key pairs for blockchain and NDN functions puts a strain on system performance.	Combining blockchain and NDN to provide secure distributed V2X communications while maintaining privacy.
[57]	Accountability	Permissioned Blockchain and Smart Contracts—PoW	Reduces message transmission time and network load	It does not meet the needs of real-time VANETs.	Event message search mechanism through blockchain and smart contracts maintaining the anonymity and accountability of VANET network users and improving the performance of the 5G network by applying SDN technology.
[58]	Accountability	Blockchain and Smart Contracts—DpoS, PoW	Small block storage pressure and high consensus algorithm efficiency	Not tested in real scenarios	A novel hierarchical certificate service chain based on blockchain for the implementation of a new reputation measurement model for effective authentication of node’s identity in VANETs
[59]	Responsibility	Blockchain—PBFT	Platform capable of providing trust to the involved entities of VANETs	There are shortcomings in the methodology as far as privacy protection is concerned	Propagation of messages based on reputation between connected vehicles and a combination of new technologies such as SDN, fog computing, and blockchain.
[60]	Responsibility	Permissioned Blockchain and Smart Contracts—PoA	The reputation score is available to individual nodes when requested with no central dependency.	The process of registering a vehicle does not guarantee concealment of the vehicle’s location	A reputation mechanism for generating, exchanging, and storing data between vehicles and of nodes in VANETs for incising vehicle and driver accountability.
[43]	Trust management	Blockchain and Smart Contracts—PoW and PoS	It provides data with integrity, security, and reliability.	Vulnerable to modeling attacks on PUFs.	Model for creating a distributed trust management system that registers and recalls vehicles using blockchain and smart contracts and the unique ID generated by the PUFs of the vehicles’ SoCs.

**Table 8:** Blockchain and smart contract methodologies against threats/attacks.

Ref	Βrute Force	Hijacking	Alteration Attack	Jamming	DDoS	Man-in-the-Middle	51% Attack Resilience	Unlinkability	Intrusion Detection	Identity Authentication	User Account Management	Tracking Attack	Sybil Attack	Location Privacy Threats	Collusion Attack	Eavesdropping Attack
[44]	✓															
[45]							✓									
[46]		✓				✓	✓	✓								
[47]												✓				
[48]															✓	
[49]			✓									✓		✓		
[50]			✓	✓								✓				
[42]										✓						
[51]			✓												✓	
[52]													✓			
[53]											✓					
[54]							✓									
[55]		✓					✓									
[56]	✓				✓					✓						
[57]			✓													
[58]						✓							✓		✓	✓
[59]					✓											
[60]															✓	
[43]			✓					✓		✓						

**Table 9:** Potential future directions of CCAM.

Future Direction	Description
Well-structured trust models	Models that will create a climate of trust and security for users and that will include all involved entities, different types of data, different properties, measurements, and parameters.
Building a framework to protect against a set of attacks	Methodologies should cover many different types of attacks and not just a few. A comprehensive framework for dealing with most VANET attacks and failures should be designed and evaluated.
Mechanisms and methodologies with a small energy footprint	Defining parameters in the processes and operations that will be implemented in order to reduce the consumption of resources that affect energy consumption and environmental pollution.
Comprehensive profiling, reputation and rating system for all entities involved	Creating a profile based on the contribution to road incident data, but also defining the reputation of each entity (fixed or not, direct or indirect), but also creating a reward system for its behavior in the road ecosystem.
Use of Federal Learning and Artificial Intelligence technologies.	Applying Federated Learning and Artificial Intelligence models to create a global intelligence in the IoT ecosystem.
Use of emerging technologies	Cloud Services, Fog, and Edge computing, Software Defined Networking (SDN), Network Functions Virtualization (NFV).
Improving the performance of blockchain technology for use in different applications	Blockchain offers users many different applications with different performance requirements, which must be met by blockchain technology in order to overcome latency and load issues.
Improvement and development of detection systems and sensors	Further research into the creation of more reliable and efficient devices and sensors, filtering and evaluating the data they produce before being sent to the RSUs and central infrastructure.
Balancing between decentralization and network load	Blockchain technology, the consensus mechanism, and the constant exchange of large volumes of data burdens the network and causes load and delays.
Allocation of resources, processing and storage	Due to the complexity and decentralization of blockchain technology, as well as the dynamic nature of the blockchain, new performance and resource allocation challenges arise.
